# Membrane Distillation of Meat Industry Effluent with Hydrophilic Polyurethane Coated Polytetrafluoroethylene Membranes

**DOI:** 10.3390/membranes7040055

**Published:** 2017-09-29

**Authors:** M. G. Mostafa, Bo Zhu, Marlene Cran, Noel Dow, Nicholas Milne, Dilip Desai, Mikel Duke

**Affiliations:** 1 Institute for Sustainability and Innovation, College of Engineering and Science, Victoria University, Werribee Campus, P. O. Box 14428, Melbourne 8001, Australia; mostafa_iesru@yahoo.com (M.G.M.); bo.zhu@vu.edu.au (B.Z.); marlene.cran@vu.edu.au (M.C.); noel.dow@vu.edu.au (N.D.); n.milne@deakin.edu.au (N.M.); 2 Institute of Environmental Science, University of Rajshahi, Rajshahi 6205, Bangladesh; 3 Pinches Consolidated Industries, 21 Malua Street, Reservoir, Victoria 3073, Australia; dkdesai@pinches.com.au

**Keywords:** stick water, meat industry, membrane microfiltration, membrane distillation

## Abstract

Meat rendering operations produce stick water waste which is rich in proteins, fats, and minerals. Membrane distillation (MD) may further recover water and valuable solids, but hydrophobic membranes are contaminated by the fats. Here, commercial hydrophobic polytetrafluorethylene (PTFE) membranes with a hydrophilic polyurethane surface layer (PU-PTFE) are used for the first time for direct contact MD (DCMD) on real poultry, fish, and bovine stick waters. Metal membrane microfiltration (MMF) was also used to capture fats prior to MD. Although the standard hydrophobic PTFE membranes failed rapidly, PU-PTFE membranes effectively processed all stick water samples to colourless permeate with sodium rejections >99%. Initial clean solution fluxes 5–6 L/m^2^/h declined to less than half during short 40% water recovery tests for all stick water samples. Fish stick water uniquely showed reduced fouling and up to 78% water recovery. Lost flux was easily restored by rinsing the membrane with clean water. MMF prior to MD removed 92% of fats, facilitating superior MD performance. Differences in fouling between stick waters were attributed to temperature polarisation from higher melt temperature fats and relative proportions to proteins. Hydrophilic coated MD membranes are applicable to stick water processing but further studies should consider membrane cleaning and longer-term stability.

## 1. Introduction

Stick water is the liquid waste produced from meat rendering, which is rich in fats, proteins, and minerals [1]. The high organic concentration of this waste means that either further site treatment is needed, for example by conventional physical and biological processes, or high prices are paid to discharge to the local sewer facility. More advanced separation processes utilising membrane technology could provide a convenient and efficient means of clean water recovery and waste minimisation [2,3,4,5,6,7,8,9]. Of the various types, a more recent membrane technology for industry application is membrane distillation (MD), which may see advantage when applied for stick water processing.

MD is a thermally-driven phase change separation process which conveniently utilises waste thermal energy from an industry site to capture clean distillate as well as concentrate wastes, including dissolved solids such as minerals which has driven the focus to desalination [5]. Further, application of MD has been shown in various industry contexts including a treatment of wastewaters at a power station [10], textile plant [11], food processing facility [12], and metal forming industry [13], as well as dairy product concentration [14]. More details of MD applications can be found in recent reviews [15,16]. One key advantage of MD in treatment of industrial wastewater including stick water compared to other processes (including other membrane processes), is its ability to highly concentrate all solids in solution (including dissolved solids), enabling the possibility for product concentration, while at the same time producing a high-quality water by-product. In a typical setup, a hydrophobic membrane allows the vapour phase to pass through the membrane’s pores while inhibiting liquid phase transport. The partial vapour pressure difference driving force is imposed by temperature gradient, where in the direct contact MD (DCMD) mode, warm solutions circulated on one side of the membrane will release clean water vapour through the membrane to a cooler permeate circulated on the other side of the membrane. Acceptable MD performance depends on membrane characteristics, i.e., pore size, pore size distribution, affinity for water, thermal conductivity, and appropriate membrane thickness [2,7,8,9].

Hydrophobic microporous membranes, such as polytetrafluoroethylene (PTFE), polyvinylidene fluoride (PVDF), polyethylene, or polypropylene (PP), can fulfil the basic requirement of hydrophobicity. PTFE has excellent thermal stability, strong chemical inertness, high mechanical strength, and great insulating performance and has consequently been widely used for MD [17,18]. However, the hydrophobic property is attractive to the hydrophobic groups of molecules present in certain solutions, causing flux decline and membrane wetting (liquid phase transport through the membrane) [19]. This is relevant in the case of fat-rich stick water. Pre-treatment to remove hydrophobic materials such as oils by gas flotation avoided these negative effects on MD membranes [20], however it may be desirable to directly process the solution without pre-treatment. Novel approaches to make the fluid compatible with the membrane to avoid wetting can involve tailoring the membrane chemistry. An example of membrane chemistry tailoring has successfully demonstrated wetting avoidance during lab MD testing of solutions containing alcohols, surfactants, or oils by novel chemistries including hydrophilic coated hydrophobic membranes [21,22], and more recently, dual chemistry hydrophobic-hydrophilic membrane materials [21,22,23,24,25]. The success of this approach therefore warrants consideration on stick water, but these membrane materials prepared in laboratories are not readily available to industry who may be interested to adopt MD technology into their processes. Meanwhile, textiles laminated with thin PTFE membranes having a layer of hydrophilic polyurethane (PU) are a well-known, readily available commercial product in water repellent breathable fabrics [26]. This advanced material utilises the hydrophobic and durable properties of porous PTFE, while the PU surface reduces contamination from oils, detergents, other surfactants, salts, etc. [27]. This well-known, practical functionality in textiles is similarly required for MD application on solutions containing fats, oils, and salt and therefore is a suitable candidate as a MD membrane in processing stick water.

The application of MD for stick water processing may be for recovery of clean water and reduction of waste volumes. However, this may occur either on raw stick water, or after first processing with membrane filtration to capture fats and proteins. Treatment of rendering wastewater has been conducted by polymer membrane ultrafiltration (UF), followed by either nanofiltration (NF) or reverse osmosis (RO) [28]. The UF stage was found to effectively remove chemical oxygen demand (COD) by 80%, while NF or RO removed COD and dissolved solids by 99%. Despite this promising result for value adding and volume reduction of waste stick water, little is understood on membrane performance where contamination of the components on the membrane (fouling) will compromise practical operation. The same researchers proposed cleaning every three days with hydrochloric acid, while to avoid using such harsh chemicals, lipase and protease enzyme cleaners have been proposed [29,30,31]. Another approach is to instead use more durable filtration membranes made from ceramic or metal. Metal membranes are known in food/beverage applications (e.g., wine processing) for their food contact suitability (e.g., made of steel or titanium), narrow filtration cut-off, ability to handle abrasive solids and ability to achieve highly concentrated solids for dewatering applications. Therefore, metal membrane MF (MMF) may be considered to durably remove fats and COD from stick water, followed by an MD stage to recover water and reduce waste volume.

The objective of this work was, therefore, to explore the application of MD to treat problematic stick water produced by the meat industry, using commercially available standard hydrophobic PTFE membranes, and the more novel hydrophilic PU coated hydrophobic PTFE (PU-PTFE) membranes. The latter is applied to prevent the attachment of hydrophobic fats to the underlying hydrophobic membrane. MD of raw stick water will be considered for direct water recovery and concentration, as well as MD of stick water pre-treated by MMF to replicate an upstream solids removal stage that may be applied in a stick water treatment process train. The findings will be used to support the case for the novel membrane chemistry in fat-rich applications to avoid the problematic wetting issue in MD, while also demonstrating the potential for MD in the treatment of problematic meat industry wastewater (stick water).

## 2. Materials and Methods

### 2.1. Stick Water Samples

The poultry (PStW), fish (FStW), and bovine (BStW) stick water samples used for the current work were taken from separate large size rendering plants based in Australia. The BStW and PStW samples were sterilised within 12 h of collection for safe handling as a feed for MD and MMF by treating at 121 °C for 40 min in an autoclave.

### 2.2. Membrane Testing

MD testing was conducted in a bench scale apparatus in direct contact MD (DCMD) mode utilising a setup (including flow schematic) reported elsewhere [14,32]. Briefly, an acrylic MD module was used with an active membrane area of 0.0163 m^2^. The hot and cold cycles where pumped using a double head peristaltic pump attached to a single drive, providing both cycles with rates of 545 mL/min, corresponding to a superficial cross flow velocity over the membrane surface of 0.10 m/s. The MD membrane used for this study was supplied by Australian Textile Mills and features a hydrophilic PU coating on top of hydrophobic PTFE (PU-PTFE) laminated on a woven fabric for support. The comparison hydrophobic membrane sample was a high performance MD membrane, similar to those utilised previously in pilot trials [10], which was supplied by Ningbo Chanqi, China, which featured a standard highly hydrophobic PTFE (standard PTFE) with nominal 0.5 μm pore size bonded to a polypropylene scrim support. The initial membrane performance for salt rejection and clean water flux, *J_CW_*, was determined by testing 1% NaCl solution using the MD rig with 60 °C hot (feed) cycle temperature and 20 °C cold (permeate) cycle temperature. The membrane testing on the stick water samples was carried out by two operations: (i) direct MD of all raw stick waters and (ii) MD of MMF pre-treated BStW. The choice of temperatures for the hot and cold side of the MD system represent a suitably high temperature available (>60 °C) at meat rendering plants as a thermal energy source transferred to MD, and ambient cooling temperatures of around 20 °C, respectively. The MD runs started with a feed volume of 1.5 L and approximately 600 mL of DI water in the cold cycle where the permeate evolves.

MMF of the stick water sample was carried out using the cross-flow metal membrane filtration unit (AMS BT500-65 filtration system) provided by Advanced Material Solutions (AMS), Lonsdale, SA, Australia. The cross-flow metal membrane rig was installed with a 0.5 μm membrane consisting of a bundle of sintered metal tubes with an active membrane area of 0.26 m^2^. Filtration was performed inside-out under an operating mode with a pressurised air activated back pulse every 3 s and a back flush every 5 min. The stick water (8 L) was charged in the feed tank prior to starting the batch run with 3.5 L of permeate collected from the stick water feed solution. The system was operated at the low pressure received from the circulation pump, measured at 17 kPa.

For MD and MMF tests, the instantaneous flux (*J*) was calculated according to:

(1) $$J=\frac{v}{At}$$

where, *v* (L) is the volume of the collected permeate over a given time period, *t* (h), per unit of membrane area, *A* (m^2^).

In order to assess membrane fouling over time on MD membranes, the overall mass transfer coefficient, *C_m_*, is a useful parameter as it removes variations in operation temperature which also influence membrane flux [10]. It therefore more clearly shows change in membrane performance due to fouling as well as changing conditions such as cycle flow rate. *C_m_* is given by [32]:

(2) $$C_{m}=\frac{J}{\Delta p_{vap}}$$

where Δ*p_vap_* is the vapour pressure drop of the bulk solutions on either side of the membrane. While this method to determine *C_m_* accounts for the difference in vapour pressure drop from the bulk solution, it is still influenced by other effects including cross flow velocity, spacer performance, and fluid properties, as the true driving force is based on the membrane surface temperatures instead of the bulk solution. The difference in temperature between the bulk solution and the membrane surface is known as temperature polarisation, and will be discussed as part of the results when presenting *C_m_*. The value of Δ*p_vap_* is therefore determined by the log mean pressure drop for counter current systems [32]:

(3) $$\Delta p_{vap}=\frac{\left(p_{h,i}-p_{c,o})-(p_{h,o}-p_{c,i}\right)}{\ln\frac{\left(p_{h,i}-p_{c,o}\right)}{\left(p_{h,o}-p_{c,i}\right)}}$$

where subscript ‘*h*’ designates the hot feed side of the module, ‘*c*’ the cold permeate side of the module, ‘*i*’ is the inlet port and ‘*o*’ is the outlet port (four ports in total). These vapour pressures were determined from the corresponding temperatures entering or leaving the membrane using the Antoine equation, which has been previously applied to determine vapour pressure differences in MD systems [32].

### 2.3. Sample Quality Analysis

Electrical conductivity (EC) of the water samples from membrane testing was determined with a portable conductivity meter (Sension156, Hach, Loveland, CO, USA). Total fat, protein, ash, and COD of the water samples were measured at a commercial laboratory, DTS Food Laboratories, North Melbourne, Australia. Fat was analysed using the Soxtec Method, protein by nitrogen measurement and multiplication by 6.25, and ash by combustion of dried solids at 550 °C. Fats were characterised using a fatty acid methyl ester (FAME) technique to measure the primary fatty acid groups present on the fats in the stick water, which was also analysed by DTS Food Laboratories, North Melbourne VIC, Australia.

Total organic carbon (TOC) and total nitrogen (TN) analysis was performed by a TOC-Vcsh TOC thermal decomposition analyser (Shimadzu, Kyoto, Japan) with an additional TNM-1 total nitrogen detector (Shimadzu, Kyoto, Japan). Sodium was determined by inductively coupled plasma-optical emission spectrometry (ICP-OES) using a Shimadzu ICPE-9000 (Shimadzu, Kyoto, Japan).

Rejection of any particular component, *i*, considered in this work, *r_i_*, was calculated by:

(4) $$r_{i}=\frac{\left(C_{i,f}-C_{i,p}\right)}{C_{i,f}}$$

where, *C_i,f_* and *C_i,p_* are measured values of the component in the feed and permeate samples, respectively. The permeate sample was measured from the sample taken from the cold cycle at the end of a batch concentration run.

### 2.4. Membrane Characterisation

#### 2.4.1. FTIR Analysis

New membranes and membranes fouled after MD processing were analysed using Fourier-transform infrared (FTIR) spectroscopy to investigate the chemical functional groups of the membranes and the subsequent attached components following MD treatment of the different feedwater samples. All membrane samples were dried at 40 °C in an oven overnight prior to FTIR analysis to remove free water that would otherwise interfere with the analysis. FTIR analysis was performed using a Perkin Elmer Frontier FTIR spectrophotometer (Waltham, USA) fitted with a diamond crystal attenuated total reflectance (ATR) accessory. Each spectrum of the membrane surface was the average of 16 scans at a resolution of 4 cm^−1^ over the frequency range 4000–500 cm^−1^ [33]. All spectra were recorded at 25 °C and the background spectra were collected and automatically deducted from sample spectra.

#### 2.4.2. SEM Imaging

Scanning electron microscopy (SEM) was employed to investigate the morphology of the membrane. The SEM images were recorded using a NeoScope JCM5000 (JEOL, Tokyo, Japan) with a 10 kV electron beam. The membranes were gold coated using a NeoCoater MP-19020NCTR (JEOL, Tokyo, Japan) prior to the observations.

## 3. Results and Discussion

### 3.1. MD Feed Sample Properties

The concentrations of various components and EC of the delivered and autoclaved (BStW and PStW samples only) samples are presented in Table 1 for each of the stick water samples provided. All had salinity indicated by EC values in the range of 13,520 μS/cm to 20,000 μS/cm as well as minerals (ash) and sodium for relevant samples. TN was relatively high, which is expected from the high protein content. All samples contained high level of organics (in terms of fats and proteins) as expected in high strength stick water effluents in meat processing plants [1]. Samples vary depending on the time at which samples were drawn from the plant, where the second BStW sample collected on another week, BStW2, contained less fats and proteins. The FStW sample had a slightly higher fat content and nearly double the protein content of PStW. All samples present different cases of stick waters that may be treated with MD. The raw stick water samples contained fats which are expected to be problematic for MD operation using conventional hydrophobic PTFE membranes but may instead be effectively processed when using hydrophilic PU-PTFE membranes.

**Figure 1:**
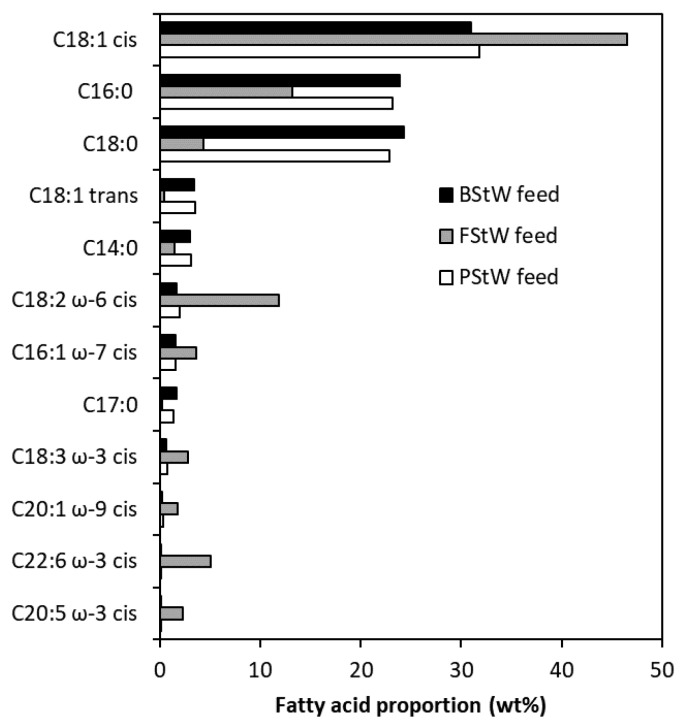
Fatty acid methyl ester (FAME) analysis result showing the weight proportion of the key fatty acid groups present in the fats from the MD stick water feed samples shown in Table 2 (PStW, FStW and BStW2). The full set of FAME analysis results are presented in Figure S1 of the Supplementary Material.

Figure 1 shows the FAME analysis results of the key fatty acid groups present in the raw stick waters. The full analysis, including trace amounts (<1%) of fatty acid groups, is given in Figure S1 of the Supplementary Material. Table 2 shows a selection of the most dominant fatty acid groups detected by FAME, including their molecular structures, relative proportions measured by FAME, and reported presence in animal derived fats and oils. All stick water samples show the strong presence of 16 and 18 carbon groups where C16:0 is palmitic acid and C18:0 is stearic acid, both saturated fatty acids. The C18:1 *cis* represents unsaturated oleic acid. All groups represent those found in lard produced from animal fats [34]. Traces of saturated myristic acid (C14:0), linoleic acid (C18:2 ω-6 *cis*), and a few others in the 16–18 carbon range were also measured. Notably, the FStW was lower in proportion of C16:0 and C18:0, and higher in proportion with C18:1 *cis* and C18:2 ω-6 *cis* fatty acid groups, with a smaller presence of individual longer chain ω-3 fatty acid groups, including docosahexaenoic acid (C22:6 ω-3 *cis*) and eicosapentaenoic acid (C20:5 ω-3 *cis*), commonly found in fish oil. The samples therefore represent the fatty acids common in animal fats (as triglycerides), which confirms the potential issues faced for hydrophobic MD membranes, as these fats would attach strongly to a hydrophobic membrane and compromise performance. 

In the case that MF may be applied upstream of the MD to remove suspended solids, the MMF BStW is also shown in Table 1 (BStW2 + MMF). MMF average flux when processing stick water was calculated to be 15 L/m^2^/h. This MMF permeate was also a feed to MD testing in this work. Fats were greatly reduced by the MMF (92% rejection) while proteins only moderately reduced (35% rejection) as the pore size of the MMF of 0.5 μm was too large to fully reject soluble proteins. This shows that fats are larger than 0.5 μm, enabling the MMF to selectively isolate most fats from proteins in the raw stick water. The fat depleted MMF permeate could then be fed to MD for the purpose of protein concentration. Previous investigations on ceramic membrane MF (CMF) of 0.17 μm on a combined effluent from a slaughterhouse measured COD and TN rejection, which were 91% and 45%, respectively [35]. These values respectively represent total fats and protein rejections, and therefore show similar separation performance to MMF. Minerals were slightly rejected by the MMF (29% rejection), indicating that a proportion of the mineral content was contained in the solids retained by the MMF. This is also supported by the drop in EC from 13,520 μS/cm to 10,280 μS/cm (Table 1).

### 3.2. MD Flux Performance

**Figure 2:**
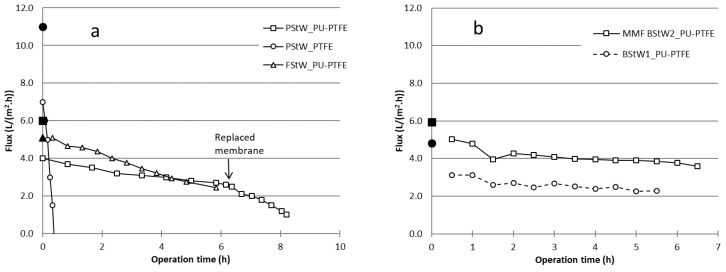
Total flux of MD tests as a function of time for PStW and FStW (**a**), and BStW (**b**) samples. Open symbols show sample MD test results, and solid symbols on *t* = 0 axis show initial clean water flux. MD hot (feed) cycle temperature = 60 °C, cold (permeate) cycle temperature 20 °C, cycle flow rates = 545 mL/min.

The flux results from the DCMD testing carried out on raw stick water samples are shown in Figure 2a for PStW and FStW and Figure 2b for BStW (including MMF pre-treated). Clean NaCl water solution tests shown as solid symbols on *t* = 0 axis. All tests showed a gradual decline in water flux over the testing time up to 8.0 h. Testing of the PStW on hydrophobic PTFE membrane (PStW_PTFE, Figure 2a) showed total loss of flux within 0.5 h in comparison to its initial clean solution flux of 11 L/(m^2^·h), declining to negative values. This is expected to be due to membrane wetting from fats present in the stick water, which attached to the membrane and facilitated liquid flow through the membrane. Previous work showed that oil from oily emulsions caused total flux loss during DCMD with pristine hydrophobic PVDF membranes, proposed to be due to long range hydrophobic–hydrophobic interactions [24]. The total loss of flux was further explained by the blockage of oil droplets which were larger than the membrane pores. As concluded above, fats in the stick water were larger than 0.5 μm, and can similarly be considered to be larger than the MD membrane pore size. However, in contrast to previously reported studies, the flux in the present study decreased but then also reversed such that the result is negative overall flux. We propose the negative flux occurred because of a slight hydraulic pressure differential over the membrane due to the cycle pumps (permeate pressure slightly higher than the feed pressure), which does not have any effect in MD when membranes are intact. However, due to wetting, this slight pressure caused a reverse liquid flow exceeding forward distillation flux. The standard hydrophobic PTFE membrane was, therefore, not suitable for MD processing of stick water, however, approaches are being considered by researchers to minimise wetting, such as pore size on other available membrane types [36], or more novel developed materials [37]. The focus in our work is to explore hydrophilic PU-coated hydrophobic PTFE (PU-PTFE) as a means to prevent wetting.

For all PU-PTFE membranes, performance lasted as long as the given test period (5.5 h–8.0 h), indicating effective performance with no wetting. However, not all stick waters performed similarly. PU-PTFE membrane fluxes during MD of raw PStW and BStW immediately dropped to about 60% of the clean water values (4.8 L/(m^2^·h) and 6.0 L/(m^2^·h) respectively) upon introduction of the stick water. Meanwhile FStW showed almost no change between the first sample flux and clean water flux (5.1 L/(m^2^·h)). Fouling occurring rapidly upon starting the MD process has been observed during MD of skim milk (no fats) on hydrophobic PTFE membranes [38]. While the interactions between individual components and the hydrophilic membrane surface will, however, be different in the skim milk case, it shows that differences in proportions of molecule groups in similar solutions leads to very different effects on MD membranes. After 6 h of DCMD operation, fluxes declined to 65%, 44%, and 73% of their values when initially exposed to stick water for PStW, FStW, and BStW1, respectively. Although the FStW flux declined the most, its higher initial fluxes achieved a water recovery of 43%. Less severe PStW and BStW1 flux declines occurred because overall the membrane produced less water due to lower flux, achieving water recoveries of only 26% and 38%, respectively. The initial autoclaving applied only the PStW and BStW samples is a difference to the FStW. While their properties that would cause differences to membrane fouling could be minor as they were both already heat treated by rendering process itself (~90 °C), further work is needed to confirm if the safety autoclaving heat treatment affected fouling. The main properties that differed between FStW and both PStW and BStW measured in this work are quantity of proteins (Table 1) and different fatty acid groups (Figure 1). How these may have altered flux due to their relative attraction to the membrane surface will be considered later in analysing the membranes post-MD testing.

As shown in Figure 2b, the flux during MD of the BStW2 pre-treated by MMF (MMF BStW2_PU-PTFE) was much higher, starting at 5.0 L/(m^2^·h) and declining to 3.6 L/(m^2^·h) (65% of the clean water flux value). This performance is far superior to raw stick water MD, and the higher flux led to a recovery of 63% over the same experiment time. The reason for the better MD fluxes on stick water when first filtered by MMF may be due to reduced membrane fouling and/or improved flow dynamics as the solution contained no suspended solids. Increasing the concentration of solids as the MD process concentrates the initial feed has been directly attributed to flux reduction, where tests on skim milk and whey showed significant flux decline as solids concentration increased [14]. To explore this more closely, the PStW MD test was extended but with a new membrane (noted as “Replaced membrane” on Figure 2a). The flux which ended at 2.6 L/(m^2^·h) continued with the new membrane at 2.5 L/(m^2^·h), following the same (but accelerated) flux decline path. This suggests that the flux decline observed over time is not associated with gradually accumulated fouling, but instead due to the physical properties of the solution that vary with concentration, such as viscosity, which can increase temperature polarisation and reduce flux.

**Figure 3:**
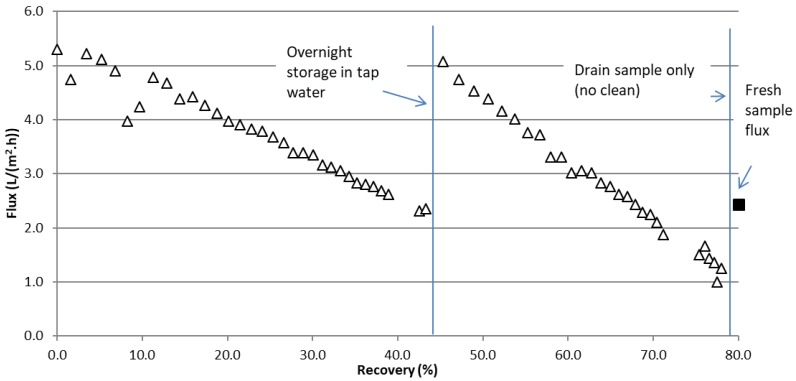
Flux during extended run on FStW using the PU-PTFE membrane as a function of water recovered from the initial sample. The solid square at end of the run is the flux when fresh sample was reloaded into the feed container.

The fouling effect, however, may be different for the FStW which did not show a sudden flux decline upon exposure to the sample. The FStW sample was therefore run for longer to further explore flux recovery and potential for high water recoveries. The results of a 2-day run on FStW are shown in Figure 3 and presented as a function of the water recovered from the initial batch. At the end of the first day (same flux data as presented in Figure 2a), the membrane module was rinsed with tap water and stored in the water overnight. Upon restarting the next day with the stick water concentrate from the previous day’s run, the flux returned to nearly the initial value (99.6% of the initial clean water flux value). Unlike PStW, the membrane fouling nearly completely reversed on a cleaned membrane, and flux proceeded to decline much like the previous day test, but at a faster rate. The fouling difference between stick waters is significant as fresh water rinsing is enough to reverse fouling from FStW components regardless of the concentrate level, while a new unused membrane installed during PStW testing instantly settled at the same flux for the given concentrate (Figure 2a).

To consider the fouling differences, the properties of the fats in the stick water are important. It is well known that oils, such as fish oils, with triglycerides consisting of unsaturated fatty acids (e.g., oleic, linoleic, linolenic) are usually liquid at ambient temperature, while tallow, such as what comes from poultry and bovine sources, with triglycerides consisting of saturated fatty acids (e.g., myristic, palmitic, stearic) are usually solid at ambient temperature [34]. Although the feed was at 60 °C, where fats are expected to be liquid, the cold side of the membrane was 20 °C where saturated fats may solidify due to the effect of temperature polarisation on the membrane feed side surface creating a temperature lower than that of the hot feed. The effect of cooling of the membrane surface on the feed side was observed in previous work on skim milk and whey, where lower fluxes at lower permeate temperatures was found to be due to stronger adhesion of proteins [14]. Therefore, it is concluded here that the above-ambient melting point of tallow from poultry and bovine sources led to the sudden loss in flux from the clean solution, and greater flux decline preventing higher water recoveries (shown for PStW during extended run in Figure 2a) in comparison to fish oil which is liquid at ambient temperatures.

The effective use of MD on PU_PTFE on stick waters, however, was only conducted up to 2 days. The fat-resistant property of the PU coating on the PTFE membrane, or other new membranes with improved chemistries or optimal properties, needs to be tested over longer operational periods in future work in order to continue demonstrating practical viability. In comparing to previous work, tests of MD in the presence of oils using oil-resisting chemistry membranes have shown promising work for crude oil emulsions [24] and mineral oils in NaCl solution [22], but at much lower concentrations using low melting point oils (liquid at room temperature). Stick water has much higher oil (as fats) content but also contains high levels of proteins, making the findings here unique.

To explore the fouling more closely, the mass transfer coefficient *C_m_* shown in Table 3 was used to compare membranes. The *C_m_* was calculated from clean solution (1% NaCl solution) as well as after initial exposure to stick water (within 30 min) and after 5 h operation. The standard PTFE membrane showed an initial clean solution *C_m_* value of 91 L/(m^2^·h·bar), which is lower than previously reported values [32] which may be due to lower cross flow velocities used here (0.1 m/s). Meanwhile, the PU-PTFE membranes all showed an initial clean solution *C_m_* varying between 37 L/(m^2^·h·bar) and 49 L/(m^2^·h·bar). This variation could be due to differences in membrane property during its fabrication. However, these values are nearly half the PTFE membrane. This suggests that the membrane could conduct only half the amount of water for a given temperature difference (vapour pressure difference). This may be due to the PU coating on the surface, different PTFE properties, and/or differences in the support layer which have a strong influence in the membrane flux [32]. A dedicated study on the role of the PU coating on heat transfer for this membrane compared to the standard PTFE as part of future work would, therefore, be useful in optimising membranes for MD application. Indeed, researchers are exploring means to produce high flux membranes with dual hydrophobic/hydrophilic layer chemistries [39], which may lead to further flux improvements in practical applications.

Looking at the standard PTFE membrane upon initial contact to PStW, *C_m_* dropped by 36% upon initial contact, then could not be calculated after 5 h operation as it was severely wetted. PStW and BStW1 samples also showed declines upon initial contact to stick water on PU-PTFE around 30%. Following the flux results in Figure 2, FStW showed almost no decline in *C_m_* upon initial contact while the MMF pre-treated BStW declined by only 16%. Despite these initial differences, after 5 h processing all stick waters without MMF pre-treatment, fouling from the stick water on the PU-PTFE membrane showed relative *C_m_* reductions in the order of 50% compared to the clean solution values. Meanwhile, in our work shown here, the most superior fouling conditions were for the MMF pre-treated stick water, which saw a reduction in mass transfer of only 38% of the clean solution value despite achieving a relatively higher recovery.

### 3.3. MD Separation Performance

**Figure 4:**
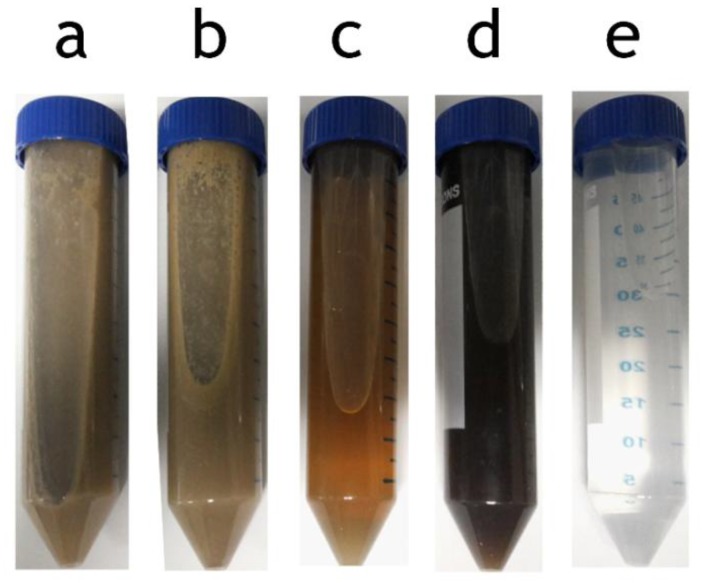
Water samples from MMF and MD of bovine stick water (BStW2): (**a**) stick water feed; (**b**) concentrate from MMF; (**c**) permeate from MMF (used as feed for MD); (**d**) concentrate from MD; (**e**) permeate from MD.

Figure 4 shows an example of the physical appearance of the samples collected from the BStW2 MMF and MD testing. It can be seen that there were significant changes to the original stick water feed (Figure 4a) as a result of MMF and MD. MMF concentrate (Figure 4b) appeared slightly thicker, while the MMF permeate (Figure 4c) was clear and brown, showing the MMF ability to effectively remove suspended solids (which fats are part of). MD of this MMF permeate led to a much darker brown colour (Figure 4d) indicating more concentrated soluble proteins. The final permeate (Figure 4e) was clear and colourless, but it did have a slight odour. The permeate from MD of raw stick water was similarly clear and colourless (not shown). Permeates of MD, therefore, had greatly improved aesthetics, making them potentially suitable for reuse on site as recycled water.

The stick water quality parameters measured from the permeate and concentrate samples are presented in Table 4, Table 5 and Table 6 for PStW, FStW, and BStW MD testing, respectively. EC was the most sensitive measure of membrane intactness, and confirmed the lost flux measured for the PTFE membrane (Figure 2) was due to wetting, as indicated by the very high EC of 3500 μS/cm in the MD permeate as a result of some of feed liquid transferring into the permeate. Fat and protein in the permeate of the standard PTFE membrane were present due to this feed liquid transfer. Meanwhile, EC measured in all cases of the PU-PTFE membranes were less than 300 μS/cm confirming that these membranes did not wet and performed well as MD membranes in the present conditions. More detailed analysis on PStW samples of MD permeates showed 99% or greater rejections of fats and proteins. Meanwhile, FStW showed equally high rejections for the same parameters except for fats, which was 88.3%. The fat in the concentrate was significantly higher for FStW due to the higher recovery achieved (78%) which may have caused some fat breakthrough.

Volatile components transporting through an intact non-wetted membrane can also contribute to permeate EC [10,11], so intactness of the PU-PTFE membranes is confirmed by high (>99%) rejection of the non-volatile sodium (Table 4 and Table 5). The standard PTFE membrane, however, showed an increase in sodium by more than one order of magnitude of the original permeate water which is strong evidence to support the conclusion this membrane had indeed wetted. TOC and TN further indicate the nature of the molecules (including volatile) that evolved into the permeate and contributed to the EC rise. TN of the PU-PTFE membrane permeate was also very low (0.04 g/L) in the permeate after processing PStW (Table 4). This suggests the EC rise was not due to ammonia as has been observed elsewhere [10]. TOC (or COD in the case of FStW in Table 5), however, was relatively high in the permeate suggesting some volatile organics were captured in the permeate. Volatile organics present in stick water which can exist in ionic form in water are volatile fatty acids (fatty acids with less than 6 carbons). FAME analysis in this work neither was unable to detect fatty acids with less than C8, nor could it analyse MD permeates due to the very low total fat quantity. However, due to the unpleasant odour of the permeate, these fatty acids are likely to be propionic acid, butyric acid, and/or 3-methylbutanoic acid. Regardless of the transfer of volatile organics into the permeate, EC still gave a good indication of membrane intactness, which is also shown in bovine stick water (BStW) as presented in Table 6.

### 3.4. Membrane Characterisation and Fouling Analysis

#### 3.4.1. SEM Imaging of Membranes Prior to Fouling

**Figure 5:**
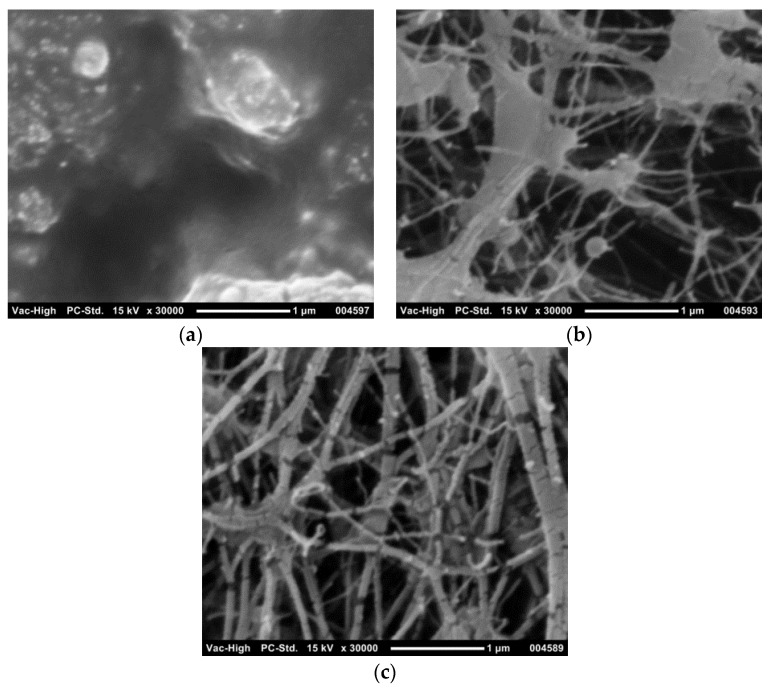
Scanning electron microscopy (SEM) image of the PU-PTFE membrane top surface (**a**) and bottom surface (**b**), and of the standard PTFE membrane top surface (**c**).

The PU-PTFE membrane was first analysed for its physical properties by SEM, as shown in Figure 5. The feed and permeate exposed PU-PTFE membrane surfaces are shown in Figure 5. The PU coating (applied to the membrane feed) showed no observable pores (>50 nm) at the same scale as the bottom side (applied to the permeate), which showed a very open fibrous structure. The standard PTFE membrane top surface is also shown, and was similar to the bottom side of the PU-PTFE membrane, which exhibited the typical fibrous structure of PTFE membranes [32]. The spaces between the fibres varied between 0.2 μm and 1.0 μm for both membranes, where the size range for the standard PTFE membrane is in agreement with the suppliers nominated pore size of 0.5 μm. The PU-PTFE’s non-laminated bottom side contained larger solid nodes, but fibres were thinner and spaces appeared larger than standard PTFE, suggesting the presence of larger pores up to around 1 μm in the PTFE layer of this membrane.

#### 3.4.2. Visual Observation of Fouling

**Figure 6:**
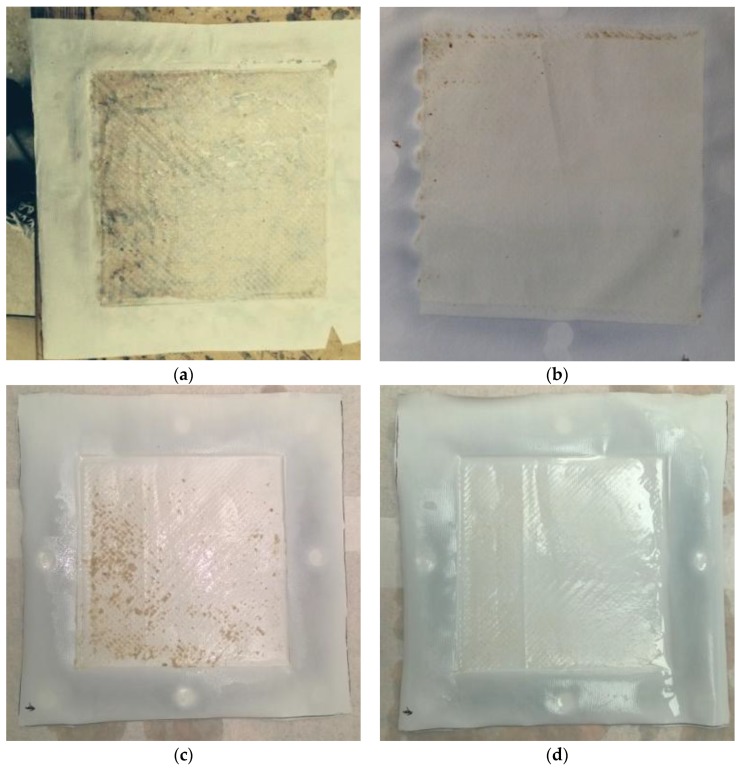
Photographs of membranes after treatment with PStW, showing (**a**) standard PTFE and (**b**) PU-PTFE membranes. Images show the feed contact side of the membrane, where the feed solution was fed from left to right. Photographs of PU-PTFE membrane (**c**) after MD with FStW, and (**d**) the same membrane after gentle rinse in tap water.

After MD treatment, the fouled membranes were analysed visually by direct observation and via SEM. Figure 6 shows the wetted standard PTFE and comparison PU-PTFE from MD processing of poultry stick water. The effect of wetting observed by flux (Figure 2a) and significant EC increase (Table 2) is further confirmed by the transition of the membrane from opaque white to clear colourless as a result of fats soaking into the membrane (black markings on underlying table can be seen through the membrane). The PU-PTFE membrane treating the same stick water, however, showed no similar transition to transparency but some evidence of fouling (brown deposits). Also shown in Figure 6 is the PU-PTFE membrane fouled after the extended recovery test on FStW (Figure 3). Some brown deposits were also found at the end of the run, but these were easily rinsed off with gentle tap water flow as shown.

**Figure 7:**
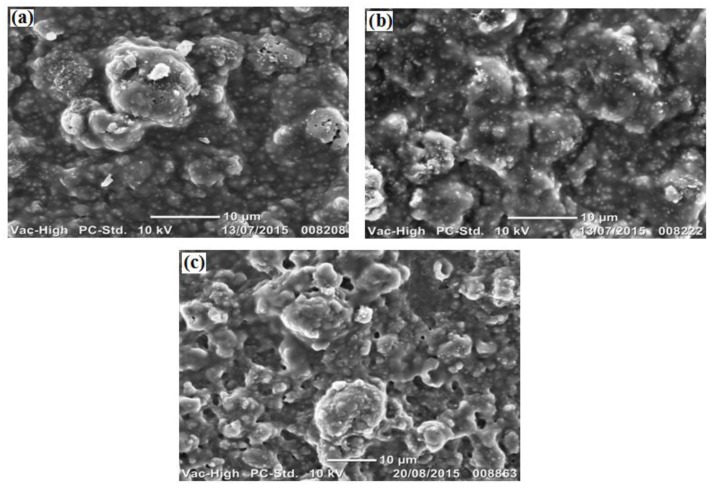
SEM images showing membrane surface of (**a**) original unused PU-PTFE; (**b**) PU-PTFE after MD with MMF filtered BStW2, and (**c**) PU-PTFE after MD with BStW1.

Bovine stick water (BStW) fouled membranes were selected for SEM analysis of microscale morphologies to explore the interactions with stick water components on the PU-PTFE membranes, as shown in Figure 7. The membrane used in the MD test fed with MMF treated stick water (Figure 7b) shows very little difference compared to the original untested membrane (Figure 7a) indicating minor attachment of fouling material as a result of MD testing. This correlates to the flux results in Figure 2b, where operation fluxes were close to the clean water fluxes, declining as concentration increased. Meanwhile, MD operation with raw stick water gave a very different surface appearance (Figure 7c), where material with some pores around 1 μm was clearly attached to the surface. However, the attachment was not uniform, as the spots identified on the original unused membrane between the protruding attachments were still evident.

#### 3.4.3. FTIR Analysis

**Figure 8:**
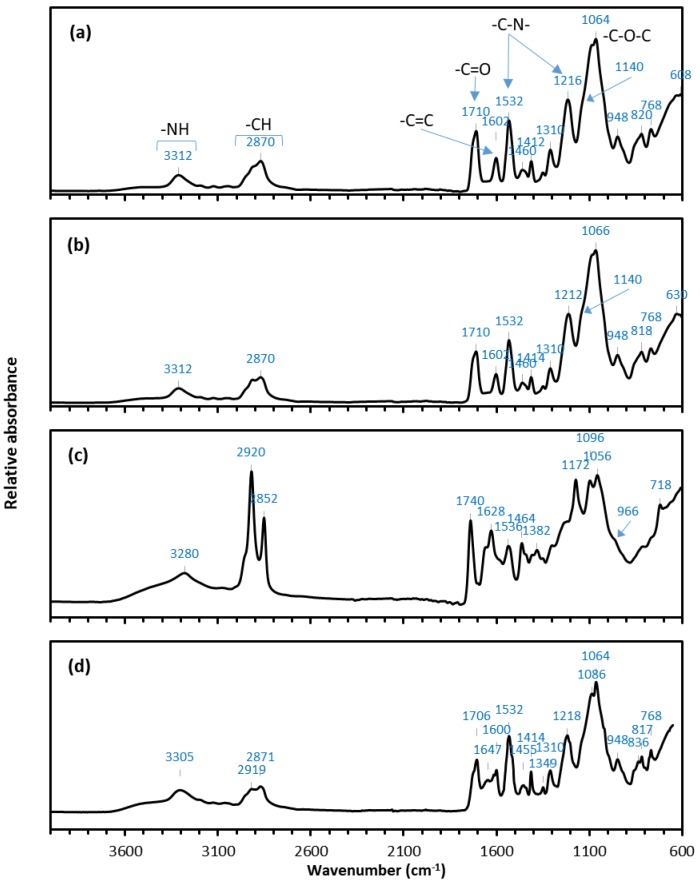
Fourier-transform infrared (FTIR) spectra of (**a**) unused PU-PTFE membrane sample; (**b**) PU-PTFE fouled during MD with MMF treated BStW2; (**c**) PU-PTFE fouled during MD with raw BStW1, and (**d**) PU-PTFE fouled during MD with raw FStW.

FTIR analysis was performed on representative samples to identify chemical functional groups on the new and fouled membranes. This helped elucidate differences between the major foulant species relative to the bovine (BStW1) and fish (FStW) stick water feed samples which had very different properties, as shown in Table 1 and Figure 1. Figure 8 shows the FTIR spectra of the unused PU-PTFE membrane, the PU-PTFE membranes fouled with MMF treated BStW2 and raw BStW1 during MD processing. The unused membrane spectrum shown in Figure 8a is characteristic of aromatic PU with a broad –NH peak at 3300 cm^−1^, –CH peaks at 2900–2800 cm^−1^, –C–N– peaks at 1532 and 1216 cm^−1^, –C=O peak at 1710 cm^−1^, and –C–O–C– peaks at 1050–1150 cm^−1^ [40,41,42,43]. The aromaticity of the PU is confirmed by the presence of the –C=C peak at 1600 cm^−1^ [44]. Although overlapped by the –C–N– PU peaks, the PTFE layer under the PU coating may also contribute to the peak at 1216 cm^−1^ where the –CF_2_ vibrations arise [45]. Additional evidence of the PTFE layer may be observed by the slight shoulder in the region of 1140 cm^−1^ where further bending vibrations of –CF_2_ occur.

The spectra shown in Figure 8b representing the MMF treated BStW2 fouled membrane is very similar to that of the unused PU-PTFE membrane shown in Figure 8a. This supports the previous flux (Figure 2b) and SEM (Figure 7b) results indicating that fouling was minimal on the membrane used for the MD operation on MMF permeate. However, several new peaks are evident on the raw BStW1 fouled membrane as shown in Figure 8c. The increased intensity and broadened peak at around 3280 cm^−1^ is attributed to the N–H stretch of the PU but it may also be a contribution from the amide bending vibrations from proteins in the stick water. Protein fouling is also shown by the peak at 1628 cm^−1^ which is representative of N–H amide I groups, as well as the peaks at 1740 cm^−1^ and 1382 cm^−1^ which are due to the stretching and bending vibrations of the C–O bonds in amino acids (–C=O–OH) [46]. The intense peaks at 2920 cm^−1^ and 2852 cm^−1^ show the presence of aliphatic –CH groups that are evidence of lipids or fats on the membrane surface. The peaks at 1172 cm^−1^ and 719 cm^−1^ also show the presence of C–H bending of fatty esters. The shoulder on the peak at 966 cm^−1^ corresponds to the *trans* double bond in fatty acids which is further evidence of the presence of fat on the membrane surface. Thus, the FTIR results show that both fatty acid esters and proteins from the raw BStW1 adsorbed onto the surface and subsequently fouled the PU-PTFE membrane. 

Dairy proteins in the absence of fat interacted strongly with hydrophobic PTFE during MD with 5 °C permeate cycle, leaving fouling layers noticeable by SEM [47]. In the case of the hydrophilic laminated membrane and 20 °C permeate in our MD testing on stick water, the FTIR data shows both fats and proteins in the raw BStW1 strongly associated with the membrane surface. This aligns with the sudden loss in flux during MD processing uniquely for BStW1 but also likely for PStW. The FTIR spectra of the fouled PU-PTFE membrane used to treat the FStW shown in Figure 8d shows no measurable fats detected in the aliphatic –CH groups at 2919 cm^−1^ and 2871 cm^−1^, suggesting that the lack of fats attached to the membrane, as explained earlier due to different lower melting temperatures, may explain why no sudden flux drops occurred in a similar way to the fat depleted MMF pre-treated sample. More effective operation for processing poultry and bovine stick waters may be possible with elevated permeate temperatures (e.g., 30 °C), and likewise higher temperature clean water rinsing to remove foulants. Despite no measurable fats on the FStW fouled membrane, proteins were evident by the amide I (C=O) peak at 1647 cm^−1^. This protein fouling was less prominent on the FStW fouled membrane (Figure 8d) compared to the raw BStW1 fouled membranes (Figure 8c). Proteins were relatively higher than fats in the FStW (Table 1), so the unique lack of fats detected on the membrane and avoidance of sudden initial flux loss may also be attributed to the higher relative concentration of proteins in the raw FStW.

Regardless, the PU coating on the PU-PTFE was clearly an essential feature that enabled it to operate in a solution that is clearly not possible with the standard hydrophobic membrane types used more widely in MD. Future work should explore the longer-term operation of the PU-PTFE membrane and performance after regular cleaning routines.

### 3.5. Concept of MD Membrane Fouling and Its Resistance by PU-PTFE

**Figure 9:**
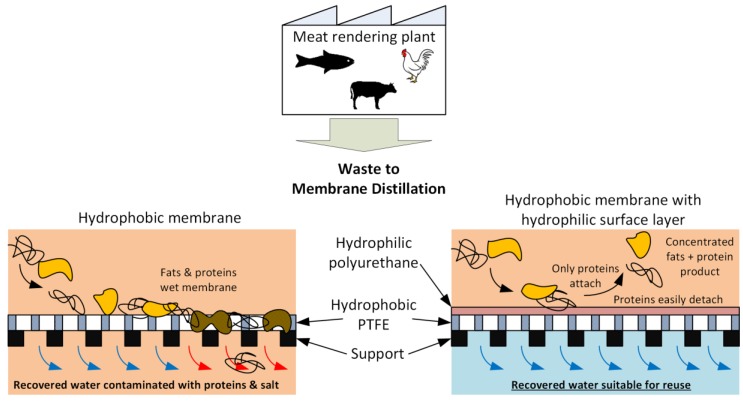
Concept of meat rendering stick water effluent fouling on the hydrophobic PTFE membrane and the fouling resistance of the hydrophilic coated PU-PTFE membrane based on results from this study.

Based on the result from this work, the concept of fouling on the hydrophobic PTFE membrane and the resistance to fouling by the PU-PTFE is shown in Figure 9. The hydrophobic PTFE membrane is shown to attract fats to the surface which compromises liquid retention leading to wetting where the liquid feed passes the membrane into the permeate. Meanwhile, the hydrophilic coating of the PU-PTFE membrane resists wetting of the underlying PTFE by fats. Although proteins can attach to the surface, they are easily removed (especially in the case of FStW).

## 4. Conclusions

MD is a wastewater processing technology that may be applied for water recovery and waste minimisation approaches to stick water from the meat processing industry. It may be applied directly to stick water, or after filtration by durable MMF. The results illustrated that the higher concentration of fats in raw stick water strongly attached to the hydrophobic PTFE membrane, and thus problematic for MD operation leading to rapid wetting and flux loss. Meanwhile, the novel application of the PU coated PTFE membrane showed no wetting and operated effectively on raw stick water over the full test duration. The hydrophilic PU coating was an effective barrier between the hydrophobic fats in the stick water and hydrophobic PTFE within the membrane. MMF prior to MD rejected fats by 92% and proteins by 35%, highlighting the potential for MMF for fat recovery prior to MD. The MD can operate to concentrate soluble proteins, while benefiting from enhanced flux performance as a result of removed fats and protein solids. The reduced flux loss from fouling which instead can operate for protein concentration.

Overall, the hydrophilic coating has clearly been advantageous to mitigate fouling in fat- and protein-dominated stick water solutions. However, the FTIR data shows that fats and proteins in the raw bovine stick water associated with the membrane surface, which aligns with the flux losses observed during MD operation. Fish stick water with higher protein and lower saturated fat uniquely showed no sudden initial flux loss and higher achievable recoveries which is likely due to the lower melting point of fish oils and/or higher protein proportion. This work has therefore found that MD application may extend into challenging industrial wastes containing fats in the meat industry with the correct selection of the membrane chemistry, membrane pre-treatment, and/or feed solution property. While all stick water feed solutions showed working MD operation, longer term effects are still unknown. Future work should explore the longer-term operation of the PU-PTFE membrane and performance after regular cleaning routines.

## Figures and Tables

**Table 1:** Water quality parameters of poultry stick water (PStW), fish stick water (FStW), and bovine stick water (BStW) (week 1 & 2) feed samples, including the permeate from metal membrane microfiltration (MMF) of BStW2 fed to membrane distillation (MD).

MD Feed	Total Fat (g/L)	Protein (g/L)	EC (μS/cm)	Mineral from Ash (g/L)	TN (g/L)	TOC or COD (mg/L) *	Sodium (mg/L)
PStW	14	39	20,000	–	7.75	42,600	4900
FStW	17	74	17,000	10	–	132,000	1410
BStW1	21	37	14,270	9	–	–	–
BStW2	12	26	13,520	7	–	–	–
BStW2 + MMF	1	17	10,280	5	–	–	–

Note: * total organic carbon (TOC) for PStW, chemical oxygen demand (COD) for FStW.

**Table 2:** Summary of fatty acids detected by FAME and reported presence in animal fats. Structure and reported presence details from literature [34].

Fatty Acid	Structure	Measured FAME Content	Reported Presence
Palmitic acid (C16:0)	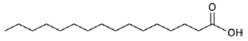	PStW = 23 wt % FStW = 12 wt % BStW2 = 23 wt %	Most of saturated fat in tallow and lard (~24%)
Stearic acid (C18:0)	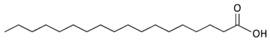	PStW = 22 wt % FStW = 5 wt % BStW2 = 23 wt %	Minor component in most oils
Oleic acid (C18:1 *cis*)	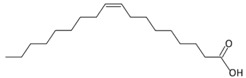	PStW = 30 wt % FStW = 48 wt % BStW2 = 30 wt %	Most widespread dietary monounsaturated fatty acid
Linoleic acid (C18:2 ω-6 *cis*)	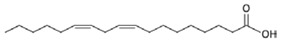	PStW = 2 wt % FStW = 11 wt % BStW2 = 2 wt %	Major polyunsaturated fat content in oil
Docosahexaenoic acid (C22:6 ω-3 *cis*)	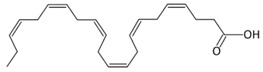	PStW = 0 wt % FStW = 5 wt % BStW2 = 0 wt %	Produced by marine algae and primary component of fish oil (8–20%)

**Table 3:** *C_m_* values calculated from clean solution testing on fresh membranes, and after 5 h of stick water processing.

Test	*C_m_* (L/(m^2^·h·bar))		
	Clean NaCl Solution	Stick Water (*t* < 0.5 h)	Stick Water (*t* = 5 h)
PStW_PTFE	91	58	Wetted
PStW_PU-PTFE	49	33	23
FStW_PU-PTFE	39	39	20
BStW1_PU-PTFE	37	24	17
MMF BStW2_PU-PTFE	45	38	28

**Table 4:** PStW MD separation results of measured components using standard PTFE and PU-PTFE membranes. Original stick water properties shown in Table 1.

Test		Total Fat (g/L)	Protein (g/L)	EC (μS/cm)	TN (g/L)	TOC (mg/L)	Sodium (mg/L)
MD run PTFE	Perm *	0.2	1	3500	0.18	391	32
MD run PU-PTFE	Conc	36.4	147	40,000	21.7	95,200	8400
	Perm *	0.2	0	260	0.04	145	3.2
	*r_i_*	98.6%	>99.9%	98.7%	99.5%	99.7%	99.3%

* Properties of the initial MD permeate are EC = 110 μS/cm, TN = 0.02 g/L, TOC = 71 mg/L and Sodium = 2.4 mg/L. Permeate sample taken at end of run prior to membrane replacement.

**Table 5:** FStW MD separation results of measured components using PU-PTFE membrane. Original stick water properties shown in Table 1.

Test		Total Fat (g/L)	Protein (g/L)	EC (μS/cm)	Minerals—from Ash (g/L)	COD (mg/L)	Sodium (mg/L)
MD run PU-PTFE	Conc	52	207	31,500	29	410,000	3840
	Perm *	2	<0.1	270	–	440	0.14
	*r_i_*	88.3%	99.9%	98.4%	–	99.7%	99.99%

* Deionised water used as initial MD permeate with EC = 10 μS/cm.

**Table 6:** BStW MD separation results of measured components using the PU-PTFE membrane, with either BStW1, or after MMF of BStW2. Original stick water properties shown in Table 1.

Test		Total Fat (g/L)	Protein (g/L)	Minerals—from Ash (g/L)	EC (μS/cm)
BStW1 MD	Conc	38	87	21	25,000
	Perm *				146
	*r_i_*				99.9%
BStW2 MF + MD	Conc	2	54	17	20,100
	Perm *				193
	*r_i_*				98.1%

* Deionised water used as initial MD permeate with EC = 10 μS/cm.

## Supplementary Materials

The following are available online at http://www.mdpi.com/2077-0375/7/4/55/s1, Figure S1: FAME analysis result showing the weight proportion of the fatty acid groups present in the fats from the stick water samples shown in Table 2 (PStW, FStW and BStW2) used as MD feeds.
