# Supplementary material for: Membrane Distillation of Meat Industry Effluent with Hydrophilic Polyurethane Coated Polytetrafluoroethylene Membranes
Source: Membranes (Basel). 2017 Sep 29;7(4):55. doi: 10.3390/membranes7040055 (PMC5746814; doi:10.3390/membranes7040055)
Supplement: Supplementary file 1 [file membranes-07-00055-s001.pdf]

Supplementary Material: Membrane Distillation of Meat Industry Effluent with Hydrophilic Polyurethane Coated Olytetrafluoroethylene Membranes

M. G. Mostafa, Bo Zhu, Marlene Cran, Noel Dow, Nicholas Milne, Dilip Desai, Mikel Duke

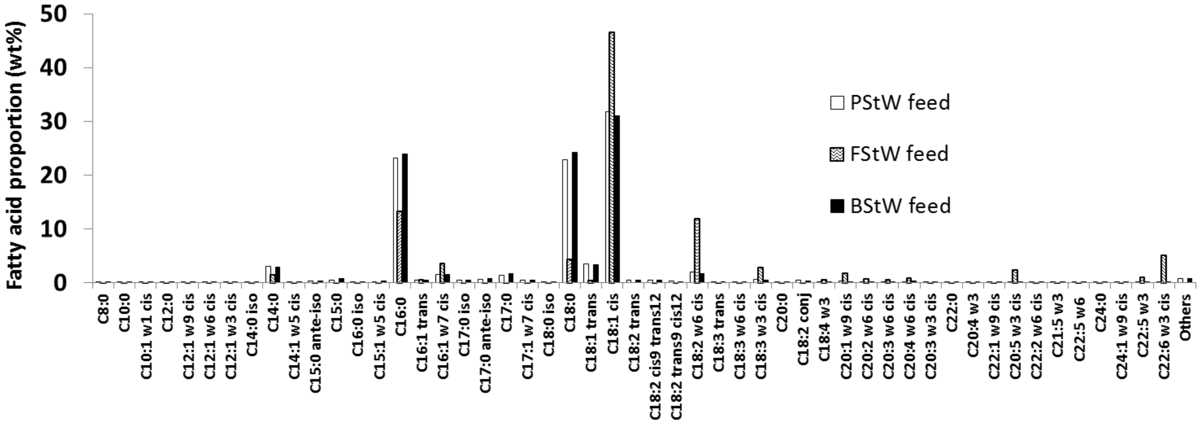

Figure S1. FAME analysis result showing the weight proportion of the fatty acid groups present in the fats from the stick water samples shown in Table 2 (PStW, FStW and BStW2) used as MD feeds.
